# Overview of 1,5-Selective Click Reaction of Azides with Alkynes or Their Synthetic Equivalents

**DOI:** 10.3390/molecules28031400

**Published:** 2023-02-01

**Authors:** Yaqi Zhao, Zhengqi Chai, Qingrui Zeng, Wen-Xiong Zhang

**Affiliations:** Beijing National Laboratory for Molecular Sciences (BNLMS), State Key Laboratory of Rare-Earth Materials Chemistry and Applications, Key Laboratory of Bioorganic Chemistry and Molecular Engineering of Ministry of Education, College of Chemistry and Molecular Engineering, Peking University, Beijing 100871, China

**Keywords:** click reaction, azides, alkynes, cycloaddition, *N*-containing heterocycles, 1,2,3-triazoles

## Abstract

Nowadays, the click reaction of azides with alkynes has evolved rapidly and become one of the most efficient methods to synthesize 1,2,3-triazoles, which are an important class of *N*-containing heterocycles. While the 1,4-selective click reaction of azides with alkynes is well established to synthesize 1,4-substituted 1,2,3-triazoles, the corresponding 1,5-selective click reaction for the generation of 1,5-substituted-1,2,3-triazoles is much less explored, and there is no systematic review for the 1,5-selective click reaction. This timely review summarizes the discovery and development of 1,5-selective click reactions of azides with alkynes for the synthesis of 1,5-substituted 1,2,3-triazoles. The 1,5-selective click reactions will be divided into three types according to the critical reactive intermediates: metallacyclic intermediates, acetylide intermediate, and formal 1,5-selective azide-alkyne cycloaddition. The related mechanistic studies will also be involved in this review.

## 1. Introduction

Click reactions are a class of atom-economical synthetic methods discovered by a group of chemists represented by K. Barry Sharpless [1]. Since the concept was presented, click reactions have received a lot of attention and have played an important role in various fields. Click reactions are an excellent category of biocompatible reactions with high chemoselectivity, allowing only specific groups of substrates to be attached to biomolecules, and therefore they are commonly used in chemical biology splicing reactions. In 2022, Sharpless, Bertozzi, and Meldal won the Nobel Prize in chemistry for click reaction and bioorthogonal chemistry [2].

**Scheme 1:**
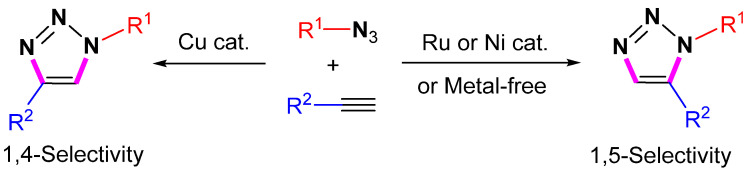
The 1,4- or 1,5-selective cycloaddition of azides with alkynes.

Nowadays, click reactions have evolved rapidly and extended to various types instead of being limited to a single specific reaction. Click reactions have also become an important tool for the synthesis of 1,2,3-triazoles with high selectivity. The 1,2,3-triazoles are an important class of *N*-containing heterocycles with diverse industrial applications as corrosion inhibitors, agrochemicals, dyes, photo stabilizers, and optical brighteners [3,4,5,6,7]. Due to their structural properties and electronic effects, they have received a lot of attention [8,9]. The Huisgen [3 + 2] cycloaddition of azides with alkynes is the most straightforward method for the synthesis of 1,2,3-triazoles. However, the reaction generally results in a mixture of 1,4-substituted and 1,5-substituted products (Scheme 1) [10,11]. The enormous synthetic potential of [3 + 2] cycloaddition reactions is limited by the obvious disadvantages, including heating requirements, prolonged reaction times, and low selectivity resulting in the formation of different isomers.

One of the most classical click reactions to form 1,2,3-triazoles is the Cu(I)-catalyzed azide-alkyne cycloaddition reaction. It can be well resolved for the highly regioselective synthesis of 1,4-disubstituted 1,2,3-triazoles with excellent yields. The use of click chemistry allows two molecular building blocks to react selectively under mild reaction conditions, forming the desired products with few or no by-products. The highly selective generation of 1,4-substituted 1,2,3-triazoles has been the focus of the studies on azide-alkyne cycloaddition reactions [12,13,14]. In contrast, studies on the generation of 1,5-substituted 1,2,3-triazole reactions are relatively rare.

**Scheme 2:**
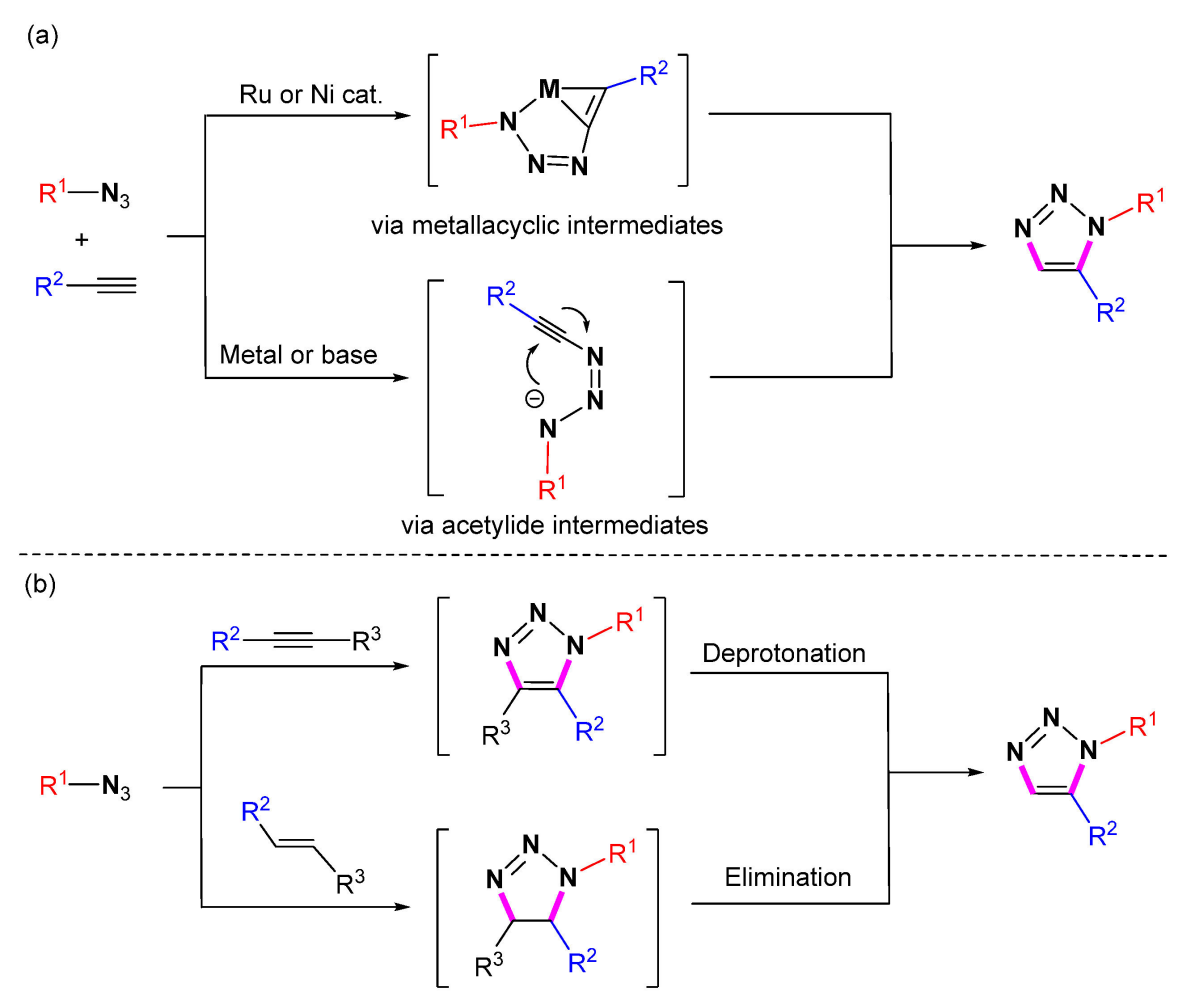
The critical intermediates in 1,5-selective cycloaddition for the synthesis of 1,5-substituted 1,2,3-triazoles. (**a**) Cycloaddition via metallacyclic or acetylide intermediates (**b**) Formal cycloaddition via metallacyclic or acetylide intermediates.

Fokin and Jia et al. discovered the synthesis of 1,5-disubstituted 1,2,3-triazoles by using ruthenium complexes to catalyze the annulation of organic azides with terminal alkynes in 2005 [15]. The full regioselective [3 + 2] cycloaddition between azides and acetylenes is possible when acetylene is activated by a strong EWG group [16]. Afterward, the syntheses of 1,5-disubstituted 1,2,3-triazoles were accomplished through many different metal-mediated or metal-free catalytic click reactions [17,18,19]. Several reviews have summarized metal-catalyzed or metal-free azide-alkyne 1,4-click reactions and the application of 1,2,3-triazoles [19,20,21,22,23,24]. Some 1,5-click reactions were sporadically incorporated into these related reviews of 1,4-click reactions. To the best of our knowledge, there is no systematic review to introduce the 1,5-selective click reaction of azides with alkynes. In this review, all the methods reported for the direct synthesis of 1,5-substituted 1,2,3-triazole compounds from azides and alkynes are described. Through this process, we have subdivided the contents into several sections according to the reaction mechanism (Scheme 2) and outlined the role of catalysts. This review aims to give a comprehensive and systematic summary of the highly selective generation of 1,5-substituted 1,2,3-triazoles.

## 2. 1,5-Selective Click Reaction of Azide with Alkyne via Metallacyclic Intermediates

### 2.1. Ruthenium-Catalyzed 1,5-Selective Click Reaction of Azide with Alkyne

#### 2.1.1. Various Reaction Conditions of Ruthenium-Catalyzed 1,5-Selective Click Reaction of Azide with Alkyne

**Scheme 3:**
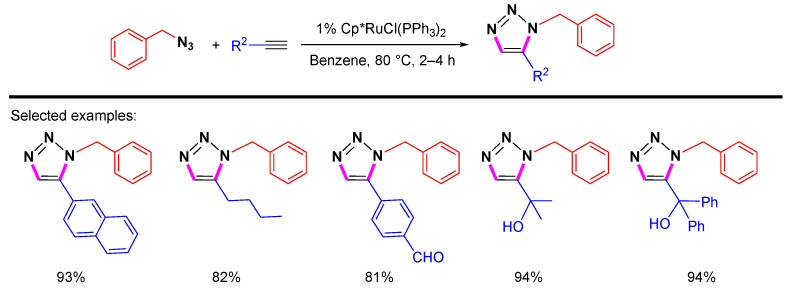
Ru-catalyzed 1,5-selective cycloaddition of benzyl azide and terminal alkynes.

In 2005, Fokin and Jia et al. first reported the synthesis of 1,5-substituted 1,2,3-triazoles via the cycloaddition reactions of azides and alkynes catalyzed by Ru-based catalysts (Scheme 3) [15]. The results show that the regioselectivity of the reactions highly depends on the ligands in the Ru catalysts. It turns out that the Ru catalysts with Cp* (Cp* = C_5_Me_5_) and Cl give better regioselectivity than those with Cp and Cl. Both aromatic and aliphatic alkynes can react with benzyl azides to give the corresponding 1,5-disubstituted 1,2,3-triazoles.

**Scheme 4:**
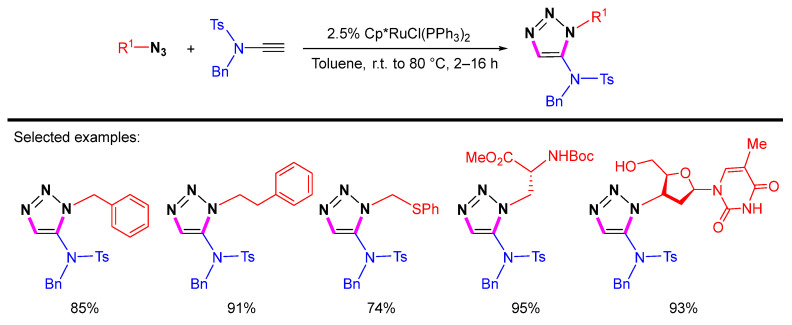
Ru-catalyzed 1,5-selective cycloaddition of azides and *N*-benzyl *N*-tosyl ynamide.

Two years later, Cintrat et al. reported that the Cp*RuCl(PPh_3_)_2_ catalyzed cycloaddition of *N*-benzyl *N*-tosyl ynamide with azides could provide 1,5-disubstituted 1,2,3-triazoles in good yields [25]. The reaction can be carried out under mild conditions to produce 1,2,3-triazoles with exclusive 1,5-regioselectivity (Scheme 4).

**Scheme 5:**
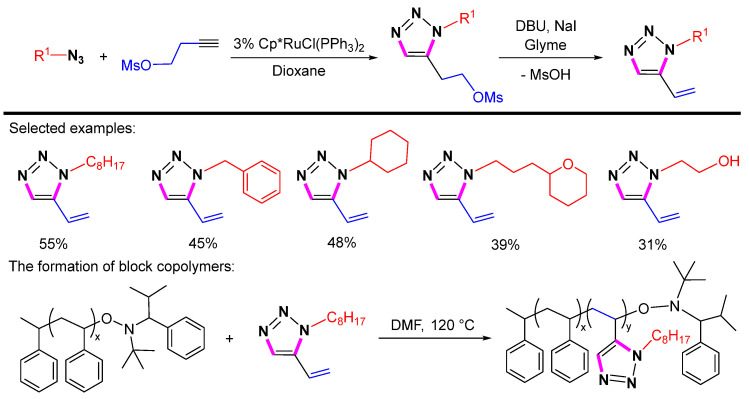
Ru-catalyzed 1,5-selective cycloaddition of azides with but-3-yn-1-yl methanesulfonate.

In 2009, Hawker et al. reported the synthesis of 1,5-substituted 1,2,3-triazoles using the same Cp*RuCl(PPh_3_)_2_ catalyst through the cycloaddition reaction of different azides with but-3-yn-1-yl methanesulfonate (Scheme 5). Subsequently, 1,5-substituted 1,2,3-triazoles can be converted into 1-substituted-5-vinyl-1,2,3-triazoles by the elimination of methanesulfonic acid in the presence of sodium iodide and 1,8-diazabicyclo [5.4.0] undec-7-ene (DBU). The 1-Substituted-5-vinyl 1,2,3-triazoles can act as monomers for living free radical polymerization, which has the potential for imparting tunable properties in polymeric materials [26].

**Scheme 6:**
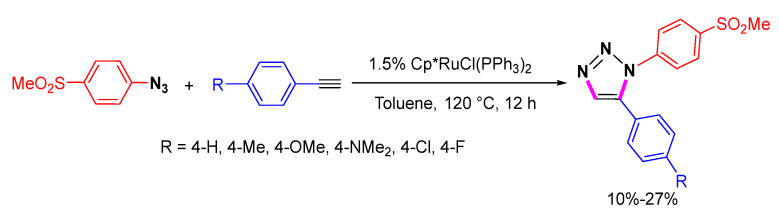
Ru-catalyzed 1,5-selective cycloaddition of 4-MeO_2_S-substituted aromatic azide with various phenyl acetylenes.

The 1,2,3-triazoles possess good properties for medicinal chemistry. Wuest et al. synthesized a series of (aryl-1,2,3-triazole-1-yl)-methanesulfonylphenyl derivatives by Cp*RuCl(PPh_3_)_2_-catalyzed cycloaddition of 1-azido-4-methane-sulfonylbenzene and para-substituted phenyl acetylenes (Scheme 6) [27]. This kind of 1,2,3-triazole can be used as in vitro cyclooxygenase (COX) inhibitors.

**Scheme 7:**
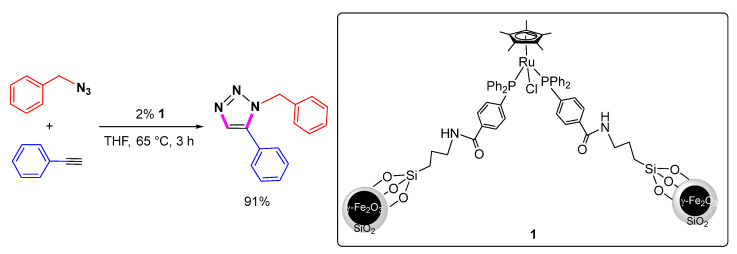
Heterogeneous Ru-catalyzed 1,5-selective cycloaddition of benzyl azide with phenyl acetylene.

In addition to homogeneous Cp*RuCl(PPh_3_)_2_ catalyzed 1,5-selective cycloaddition of azides with alkynes, the corresponding heterogeneous 1,5-selective cycloaddition was also investigated. In 2013, Astruc et al. designed and synthesized Si(OMe)_3_-functionalized triarylphosphine and immobilized Si(OMe)_3_-functionalized triarylphosphine coordinated Ru(II) complexes into oxide magnetic nanoparticles. They found that the immobilized heterogeneous Ru(II) catalyst **1** can efficiently realize the 1,5-selective cycloaddition of benzyl azide with phenyl acetylene (Scheme 7) [28]. Catalyst **1** can be recovered by simply applying an external magnetic field using a magnetic carrier. It can be recycled at least five times with only a slight decrease in catalytic activity and selectivity, making it the first recyclable catalyst for the Ru-catalyzed 1,5-selective cycloaddition.

**Scheme 8:**
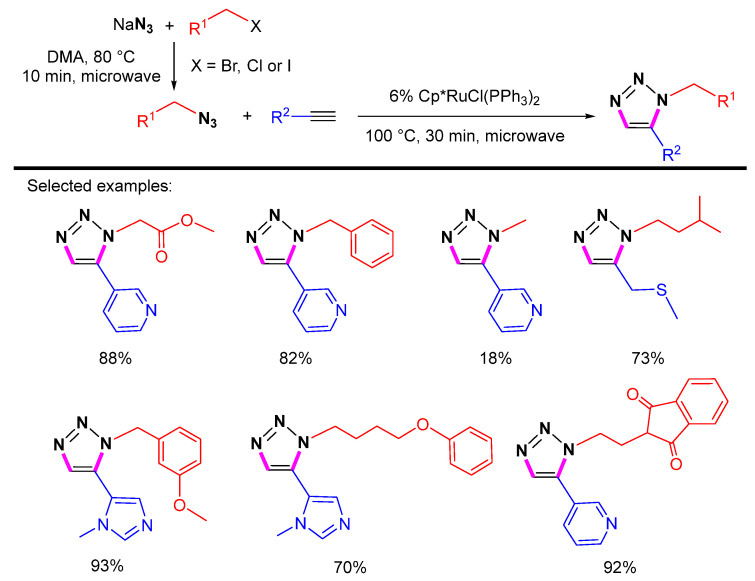
Sequential one-pot Ru-catalyzed 1,5-selective cycloaddition of alkyl halides, sodium azide, and terminal alkynes.

Organic azides with low molecular weight are considered to be highly energetic and pose an explosive risk. To avoid handling the dangerous alkyl azides, Kann et al. reported a one-pot method of alkynes with alkyl azides generated in situ from primary alkyl halides and sodium azide (Scheme 8) [29]. Under microwave irradiation, the Cp*RuCl(PPh_3_)_2_-catalyzed 1,5-selective cycloaddition can work smoothly.

**Scheme 9:**
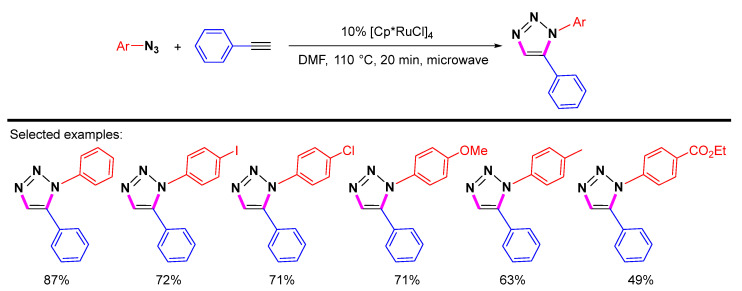
[Cp*RuCl]_4_-catalyzed 1,5-selective cycloaddition of azides with terminal alkynes.

Interestingly, Fokin et al. found that the catalytic activity of ruthenium(II) tetramer [Cp*RuCl]_4_ for 1,5-selective cycloaddition was superior to that of Cp*RuCl(PPh_3_)_2_ [30]. The cycloaddition reaction of most aryl azides with different substituents results in the formation of corresponding 1,5-disubstituted 1,2,3-triazoles in good yields (Scheme 9). The electron-rich and moderately electron-deficient aryl azides appear to be relatively favorable for the reaction. A shorter reaction time is required when the reaction is carried out under microwave irradiation than under normal conditions.

**Scheme 10:**
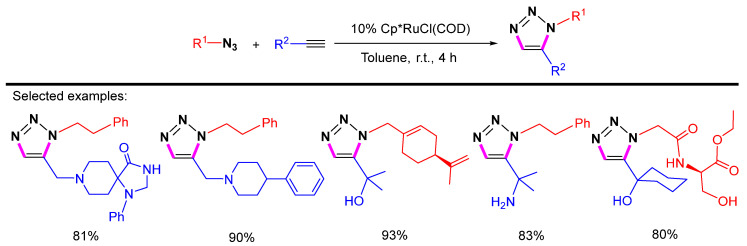
Cp*RuCl(COD)-catalyzed 1,5-selective cycloaddition of azides with terminal alkynes.

A series of ruthenium(II) complexes, such as [Cp*RuCl]_4_ and [Cp*RuCl(PPh_3_)_2_], had been reported as catalysts in azide-alkyne 1,5-selective cycloadditions. Due to synthetic availability and stability, the [Cp*RuCl(COD)] catalyst was further studied. The 1,5-cyclooctadiene (COD) ligand is more labile than other ligands, and the catalyst [Cp*RuCl(COD)] can work smoothly at room temperature [31]. Organic azides react with terminal alkynes containing various functionalities to give selectively 1,5-disubstituted 1,2,3-triazole products in the presence of a [Cp*RuCl(COD)] catalyst (Scheme 10).

**Scheme 11:**
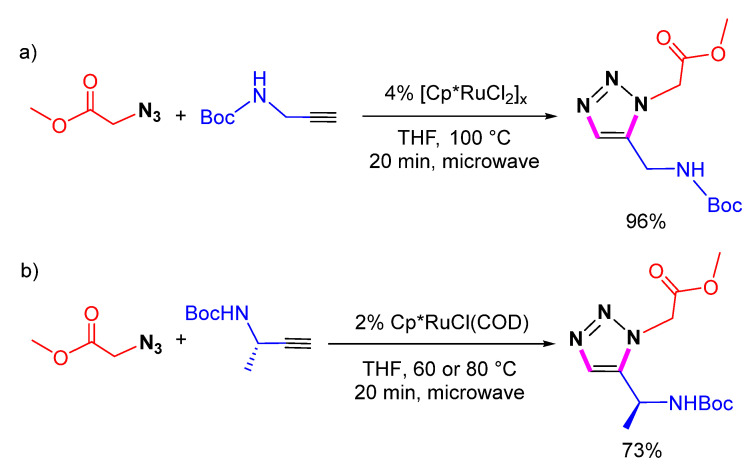
Ru-catalyzed 1,5-selective cycloaddition of methyl 2-azidoacetate (**a**) and N-Boc-propargylamines (**b**).

Tamas et al. reported the synthesis of 1,5-disubstituted 1,2,3-triazole amino acids by [Cp*RuCl_2_]_x_-catalyzed 1,5-selective cycloaddition of methyl 2-azidoacetate and *N*-Boc-propargylamine (Scheme 11a) [32]. Recently, the [Cp*RuCl(COD)]-catalyzed 1,5-selective cycloaddition of the chiral *N*-Boc-propargylamine with azide has been reported to produce the chiral 1,5-disubstituted 1,2,3-triazole amino acid (Scheme 11b) efficiently [33]. These 1,5-disubstituted 1,2,3-triazole scaffolds can be utilized to synthesize peptidic foldamers.

**Scheme 12:**
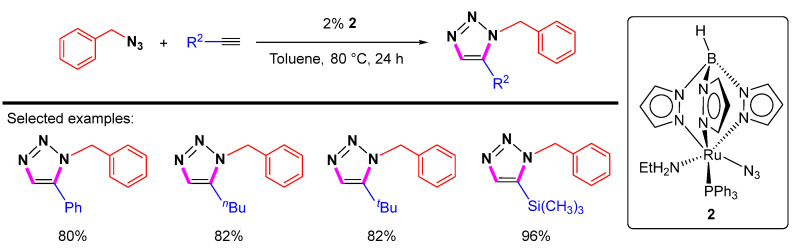
Non-Cp Ru-catalyzed 1,5-selective cycloaddition of benzyl azide with terminal alkynes.

In addition to the above-mentioned “CpRu” catalytic systems, non-Cp Ru complexes were also investigated. A series of ruthenium azide complexes containing Tp ligands were prepared. Lo et al. found the complex Tp(PPh_3_)(EtNH_2_)RuN_3_ (**2**, Tp = HB(pz)_3_, pz = pyrazolyl) is an effective catalyst for the 1,5-selective cycloaddition of benzyl azide with terminal alkynes (Scheme 12) [34]. The synthesis of 1,5-disubstituted 1,2,3-triazoles catalyzed by **2** can tolerate a number of functional groups, and the reactions can undergo in either organic or aqueous media.

#### 2.1.2. Mechanism of Ruthenium-Catalyzed 1,5-Selective Click Reaction of Azide with Alkyne

**Scheme 13:**
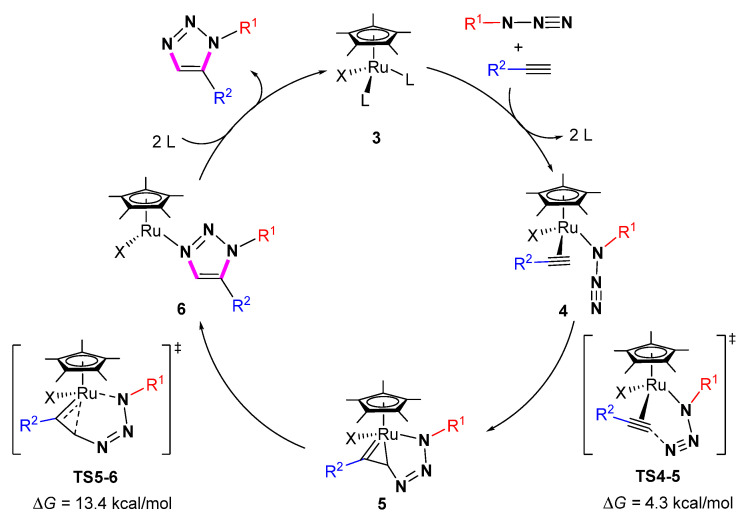
The Ru catalyzed mechanism of azide-alkyne cycloaddition.

The reaction mechanism of ruthenium-catalyzed 1,5-selective cycloaddition of azides with alkynes was summarized (Scheme 13). Firstly, the spectator ligands in **3** are replaced by azides and terminal alkynes to form the activated complex **4**. This is followed by the oxidative coupling to produce the ruthenacycle **5**. Then **5** undergoes reductive elimination to give the intermediate **6**. Finally, the dissociation of the 1,5-substituted 1,2,3 triazole and the coordination of spectator ligands in the Ru center of **6** can provide the starting **3** and complete the catalytic cycle. In 2008, Fokin and Jia et al. used DFT calculations to investigate the catalytic mechanism [31]. Computational studies indicated that the [Cp*RuCl]-catalyzed reactions of azides with alkynes involve an irreversible oxidative coupling for the nucleophilic attack of ligand alkyl groups on the terminal electrophilic nitrogen of ligand azides. The oxidative coupling step is the regioselectivity-determining step of the whole process with an energy barrier of 4.3 kcal/mol. The rate-determining step is the reductive elimination via the transition state **TS5-6** with an energy barrier of 13.4 kcal/mol.

**Scheme 14:**
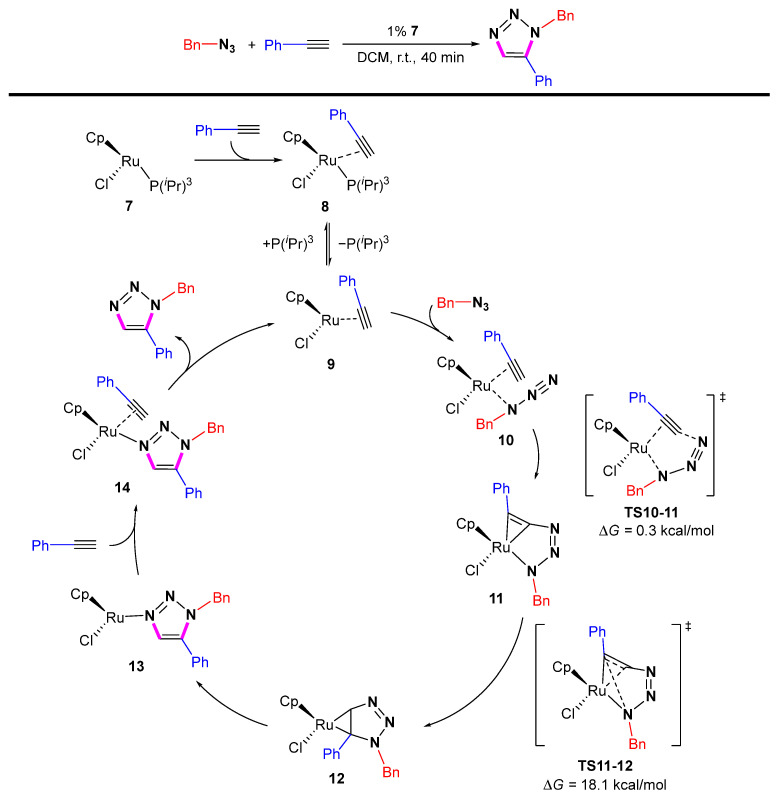
The detailed Ru catalyzed mechanism of azide-alkyne cycloaddition.

In 2012, Nolan et al. described detailed DFT studies which were in agreement with the experimental results and suggested that acetylene binding precedes azide coordination (Scheme 14) [35]. They identified previously unidentified intermediates **12** in which the formed triazole is bound to the Ru metal center in a C-Ru-C metal cyclopropane manner. Complex **12** eventually isomerizes to an *N*-bound triazole Ru species **13**. Cp*Ru(P*^i^*Pr_3_)Cl exhibits better performance than 18-electron ruthenium catalysts, allowing the production of 1,5-disubstituted 1,2,3-triazoles under mild conditions.

### 2.2. Nickel-Catalyzed 1,5-Selective Click Reaction of Azide with Alkyne

The azide-alkyne cycloadditions catalyzed by [Cp*RuCl]-based catalysts usually need to be carried out at elevated temperatures and are sensitive to water and air. Therefore, it remains a challenge to find catalysts that are compatible with water under mild conditions.

**Scheme 15:**
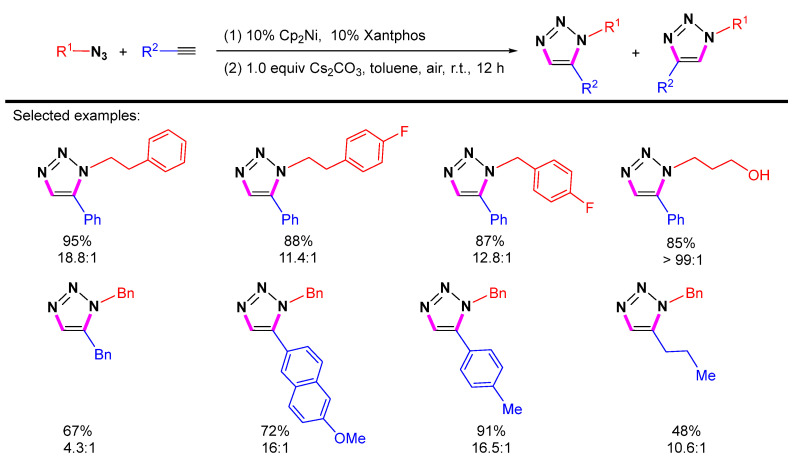
Ni-catalyzed 1,5-selective cycloaddition of azides and alkynes.

A strategy to obtain 1,5-disubstituted 1,2,3-triazoles from available substrates and inexpensive reagents via nickel catalysis in water and organic solvents at room temperature was reported by Sung et al. (Scheme 15) [36]. This nickel-catalyzed azide-alkyne cycloaddition is highly compatible with water as the only solvent and can be carried out in air at room temperature. All the substrate ranges of azides, including fluorinated aromatics and fused cyclic groups, are compatible with the reaction conditions. Both the hydroxyl and ester functional groups remain intact, and for the substrate range of alkynes, aliphatic and aromatic alkynes with different functional groups, including methoxy, amine, nitro, chlorine, and methyl, are well tolerated.

**Scheme 16:**
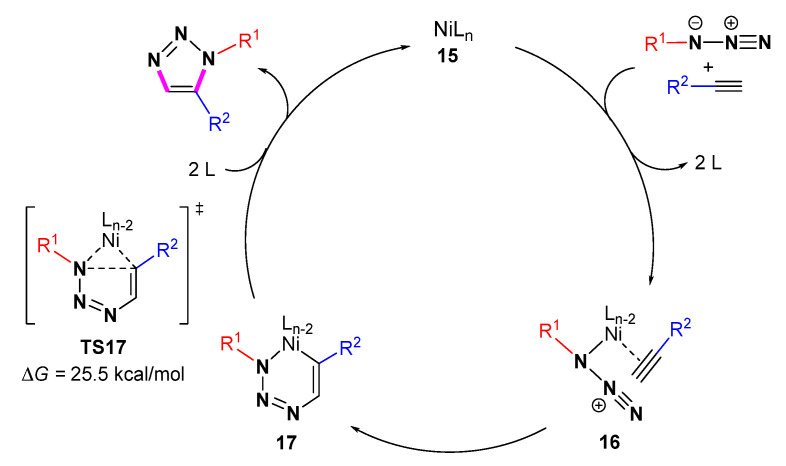
The Ni-catalyzed mechanism of cycloaddition of azides with alkynes.

They concluded the reaction involves the process of Cp_2_Ni→CpNi(Xantphos)→Ni(Xantphos)_2_ and CpNi(Xantphos)→CpNi(Xantphos)^+^. Initially, both alkynes and azides are coordinated to the Ni center to form intermediate **16**. The C-N bond formed in complex **17** between the alkyne and azide determines 1,5 regioselectivity. Subsequent reductive elimination leads to the formation of the target cyclization product and regenerates NiL_n_ species (Scheme 16). In 2020, the cycloaddition reactions of azides and asymmetric alkynes were completed under nickel catalysis [37]. DFT calculations indicate that the cyclization step via **TS17** is the rate-determining step with an energy barrier of 25.5 kcal/mol.

## 3. 1,5-Selective Click Reaction of Azide with Alkyne via Acetylide Intermediates

### 3.1. Transition-Metal-Free 1,5-Selective Click Reaction of Azide with Alkyne

**Scheme 17:**
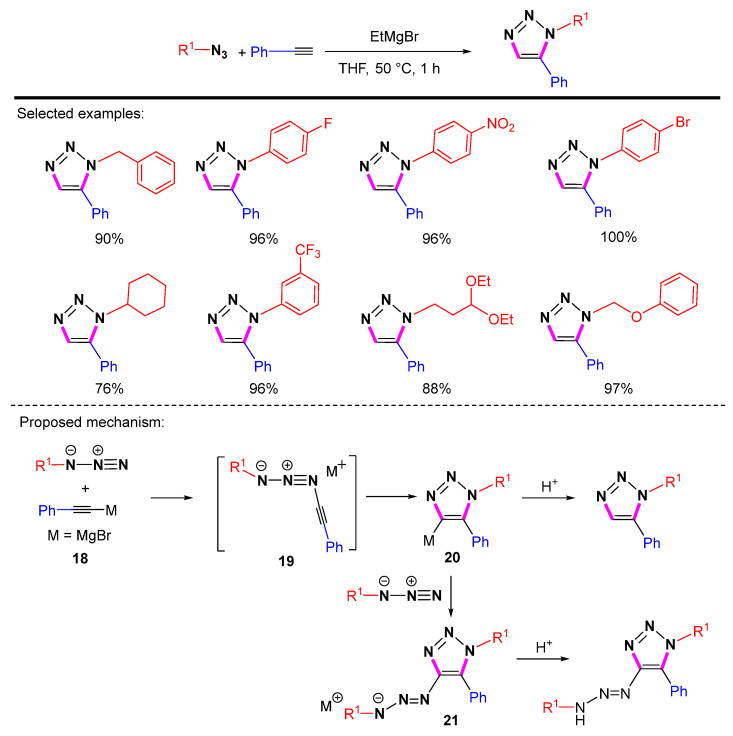
Synthesis of 1,5-disubstituted1,2,3-triazoles using magnesium acetylene reagents and proposed mechanism.

In order to control the regioselectivity of the [3 + 2] cycloaddition reaction of azides with terminal alkynes containing highly acidic C-H, a common idea is to form the corresponding alkynyl carbanions, which then selectively undergo nucleophilic attack toward the electrophilic end of the azides. The pioneer studies started in 1967 by Akimova et al., who used equivalent amounts of lithium reagents or magnesium acetylene reagents to react with terminal alkynes [38]. The reaction had limitations in terms of functional group compatibility and atomic economy. After the results had been dormant for 30 years, Sharpless et al. optimized the process to give a good yield and elaborated the mechanism of this reaction and by-product generation (Scheme 17) [39]. The mechanism begins with the nucleophilic attack of acetylide **18** on the terminal nitrogen atom of the azide to provide intermediate **19**. Then the cyclization process in **19** occurs to generate intermediate **20**. The 1,5-disubstituted 1,2,3-triazoles can be obtained by hydrolysis. Sharpless et al. concluded that, after the formation of cyclic intermediate **20**, it could be captured with electrophilic reagents to obtain the substituted 1,2,3-triazoles.

**Scheme 18:**
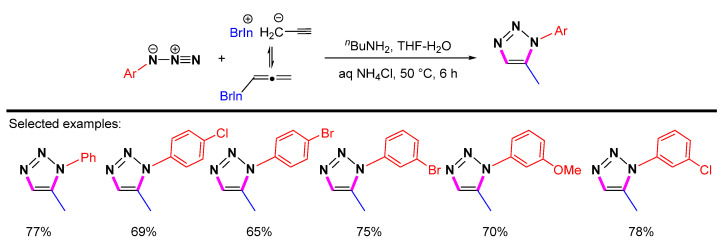
Synthesis of 1,5-disubstituted1,2,3-triazoles from allenylindium bromides with azides.

As shown in Scheme 18, the regioselective synthesis of 1-aryl-5-methyl-1,2,3-triazoles can be achieved through N/C-heterocyclization of allenylindium bromide across aryl azides [40]. The reaction can take place in an aqueous medium, and the synthesis of 1,5-disubstituted 1,2,3-triazoles can be carried out under mild reaction conditions with moderate to good yields and very high regioselectivity. However, the alkyne is limited to propargyl bromide, and only methyl-substituted 1,2,3-triazoles can be obtained.

**Scheme 19:**
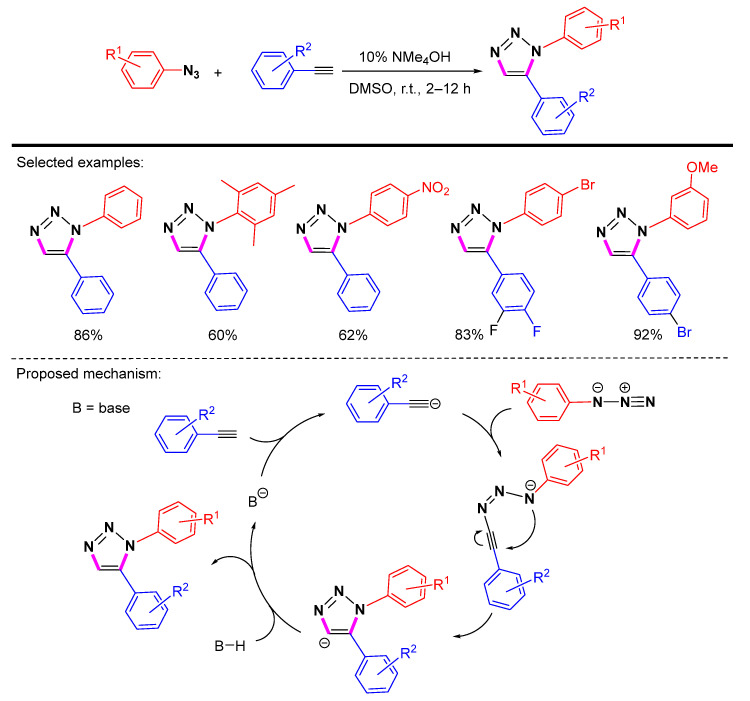
Base-catalyzed synthesis of 1,5-disubstituted 1,2,3-triazoles and proposed mechanism.

In 2010, Fokin et al. developed a base-catalyzed cycloaddition that was insensitive to both oxygen and water and did not require the involvement of metal reagents (Scheme 19) [41]. These bases, including anhydrous sodium, potassium, cesium hydroxides, aqueous tetramethylammonium, and benzyl-trimethylammonium hydroxides, can catalyze the formation of 1,5-diaryl-substituted 1H-1,2,3-triazoles. The catalytic amounts of tetramethylammonium hydroxide were used in the deprotonation of aryl acetylene to initiate the cyclic reaction. Nevertheless, the substrates for this condition are restricted to aryl alkynes, and the conversion efficiency is not very good for alkyl-substituted alkynes. The proposed mechanism shows that the reversible deprotonation of the terminal alkyne by the base produces an aryl acetylate, which then undergoes cyclization. The catalytic cycle is completed by the protonation affording the final product.

### 3.2. Zinc-Mediated 1,5-Selective Click Reaction of Azide with Alkyne

**Scheme 20:**
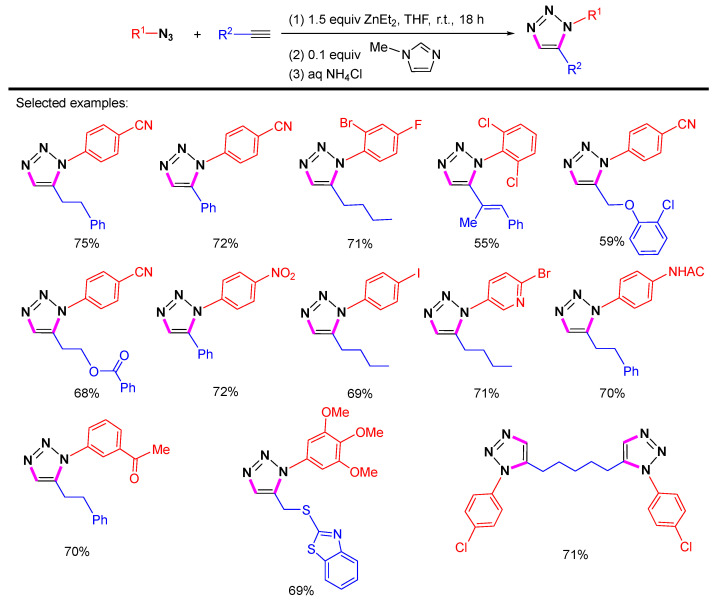
Zn-mediated 1,5-selective cycloaddition of azides with alkynes.

In 2013, Greaney et al. reported the regioselective synthesis of 1,5-substituted 1,2,3-triazoles through zinc-mediated cycloaddition at room temperature (Scheme 20) [42]. The range of alkynyl substrates includes alkyl and aryl terminal alkynes. A number of azides with different functional groups, including esters, amides, ketones, nitriles, nitros, aryl iodides, heterocycles, and ortho-ligands, can tolerate the zinc-mediated conditions. In addition, diacetylene is also suitable.

**Scheme 21:**
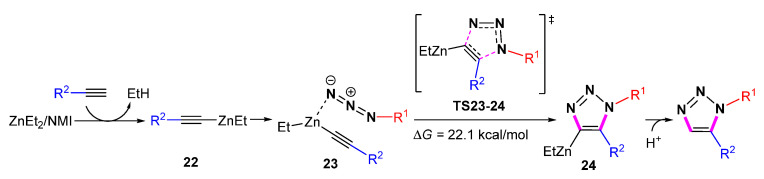
The mechanism of Zn-mediated 1,5-selective cycloaddition of azides with alkynes.

DFT calculations have been used to clarify the regioselectivity of zinc-mediated [3 + 2] cycloaddition of azides with alkynes [43]. Computational results indicate that the catalytic cycle starts with the initial metalation of the alkyne. The regioselectivity of the cycloaddition is controlled by the nucleophilicity of the terminal alkyne. The acetylide fragment is coordinated with the Zn metal center and undergoes a cyclization reaction with an energy barrier of 22.1 kcal/mol via **TS23-24** (Scheme 21).

### 3.3. Rare-Earth Metal Catalyzed 1,5-Selective Click Reaction of Azide with Alkyne

**Scheme 22:**
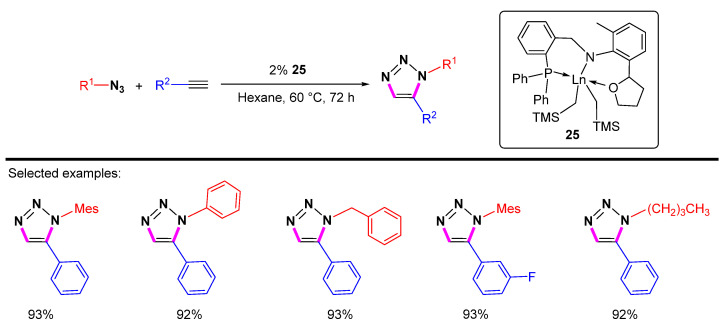
Rare-earth metal catalyzed 1,5-selective cycloaddition of azides with alkynes.

In 2008, Cui et al. reported the rare-earth metal-catalyzed cycloaddition of azides and alkynes to afford 1,5-disubstituted 1,2,3-triazoles within 72 h at 60 °C (Scheme 22) [44]. Different aromatic alkynes can be applied for the efficient synthesis of 1,5-disubstituted 1,2,3-triazoles. However, only small amounts of cycloaddition products can be obtained when aliphatic alkynes are utilized. The regioselectivity and conversion rate are also influenced by the nature of azides.

**Scheme 23:**
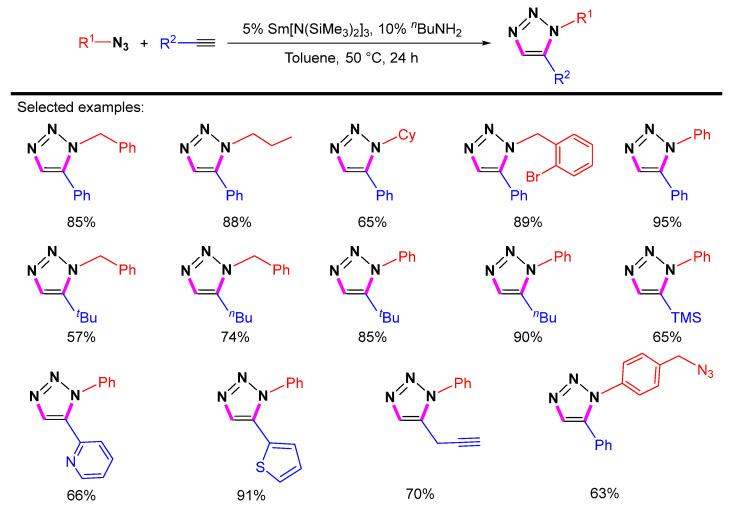
Sm[N(SiMe_3_)_2_]_3_-catalyzed 1,5-selective cycloaddition of azides with terminal alkynes.

Then Zhou et al. reported the rare-earth metal-catalyzed cycloaddition of terminal alkynes with azides to provide a series of 1,5-disubstituted 1,2,3-triazoles with good to excellent yields (Scheme 23) [45]. Catalysts containing different rare-earth metals, including Sm, Nd, Y, and Gd, have been tested, and the Sm catalyst is the best choice.

**Scheme 24:**
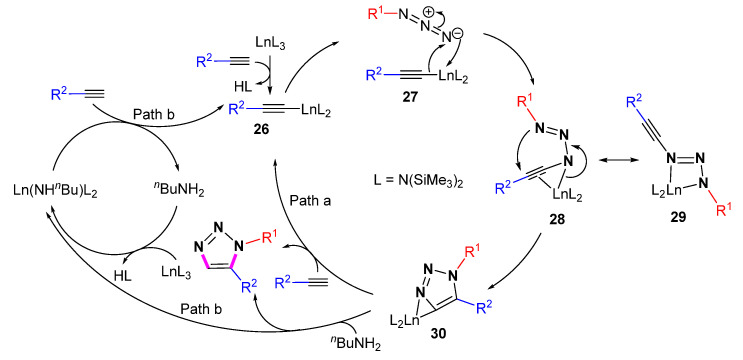
The mechanism of Sm[N(SiMe_3_)_2_]_3_-catalyzed 1,5-selective cycloaddition of azides with alkynes.

The proposed mechanism (Scheme 24) is different from the mechanism of Ru-catalyzed 1,5-selective cycloaddition. Initially, the C-H bond of the terminal alkyne can be activated to produce Ln acetylide **26**. Coordination and subsequent 1,1-insertion of azide into the Ln–C bond of **27** generate intermediates **28** or **29**. The intramolecular nucleophilic cyclization will form triazolate complex **30**. Intermediate **30** undergoes protonation with terminal alkyne to generate the 1,5-disubstituded 1,2,3-triazloes, completing the catalytic cycle. Since the amine *^n^*BuNH_2_ can act as both a proton source and ligand activation catalyst, the reaction proceeding through path b cannot be excluded.

Li et al. performed DFT calculations to investigate the mechanism of the path, namely the samarium-catalyzed 1,5-regioselective azido-alkyne [3 + 2]-cycloaddition [46]. The rate-determining step is the insertion of azide into the samarium phenylacetylide. The calculations also infer that the addition of the samarium catalyst changes the distribution of the electrostatic potential on the surface of the alkyne, determining the direction of polarization and the formation of different intermediates, which ultimately control the regioselectivity.

## 4. Formal 1,5-Selective Click Reaction of Azide with Alkyne

### 4.1. In Situ Generation of Terminal Acetylene

**Scheme 25:**
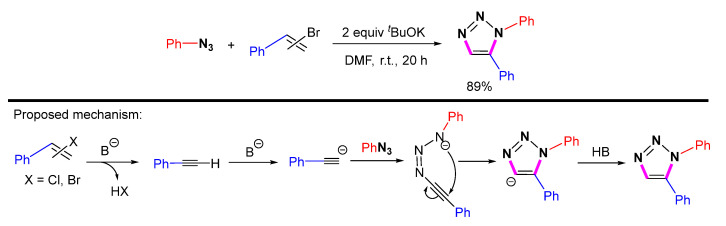
Base-mediated synthesis of 1,5-disubstituted 1,2,3-triazole from azides and vinyl bromides and proposed mechanism.

A base-mediated reaction of α- or β-vinyl bromides with azides for the synthesis of 1,5-disubstituted 1,2,3-triazoles was investigated (Scheme 25) [47]. Strong bases are necessary for the elimination of vinyl halides and the formation of alkynyl anions. Meanwhile, the reaction of aryl vinyl bromides and aryl azides tends to give high yields.

**Scheme 26:**
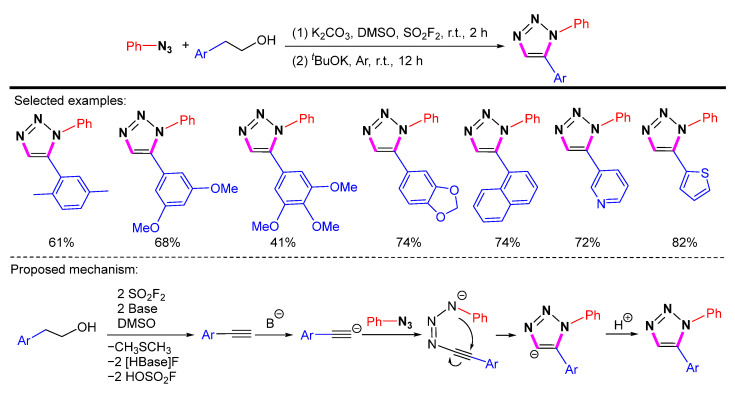
Synthesis of 1,5-disubstituted 1,2,3-triazole from azides and alcohols and proposed mechanism.

In 2019, Qin et al. found that in the presence of *^t^*BuOK, alcohols can react with azides to synthesize 1,5-disubstituted 1,2,3-triazole products in excellent yields at room temperature (Scheme 26) [48]. The mechanism of the formation of alkyne from alcohol has been reported by Qin et al. [49]. SO_2_F_2_ is used to activate DMSO for the oxidative conversion of alcohols to alkenyl sulfurofluoridates under basic conditions. Then the formation of alkynes through the elimination of HOSO_2_F in alkenyl sulfurofluoridates is promoted by the base. After transforming the alcohol into the corresponding terminal alkyne, deprotonation can occur in the presence of a strong base to generate acetylide, which reacts with the azide to afford the corresponding 1,2,3-triazole in situ. The reaction does not require a metal catalyst and shows good compatibility with a large number of functional groups.

### 4.2. Formal 1,5-Selective Click Reaction via Cycloaddition/Elimination

#### 4.2.1. Cycloaddition/Elimination of Sulfonyl Group

**Scheme 27:**
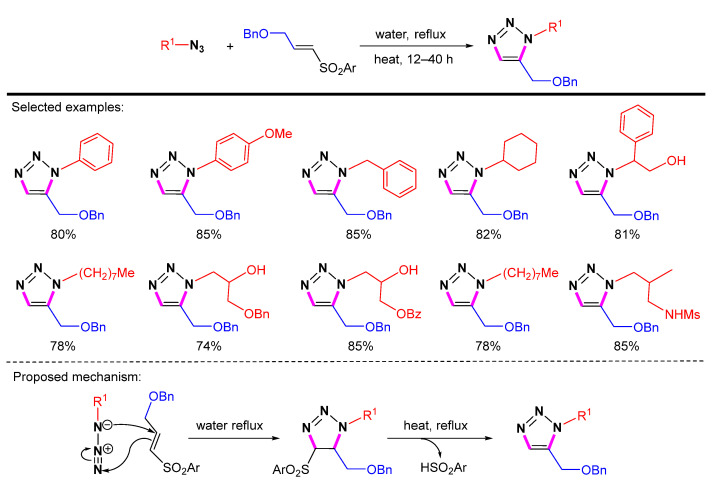
Synthesis of 1,5-disubstituted 1,2,3-triazoles from azides and vinyl sulfone and proposed mechanism.

There are few reports on the use of olefins to generate 1,2,3-triazoles in the last 20 years. The main problem is that the generation of triazolines from olefins requires additional steps of elimination or oxidation to generate 1,2,3-triazoles. In 2011, Pathak et al. reported a metal-free and vinyl sulfone-based synthesis of 1,5-disubstituted 1,2,3-triazoles (Scheme 27) [50]. This convenient and versatile process eliminates the need for an inert gas atmosphere and the use of high boiling point solvents. The strategy provides a practical route for the synthesis of 1,5-disubstituted 1,2,3-triazoles using a combination of aryl/alkyl vinyl sulfones and aryl/alkyl azides. Due to the polarization of the vinyl sulfone double bond, the azide attacks the partially positively charged position, resulting in a cyclic intermediate. The sulfinic acid is eliminated, then the 1,5-disubstituted 1,2,3-triazole is selectively produced.

**Scheme 28:**
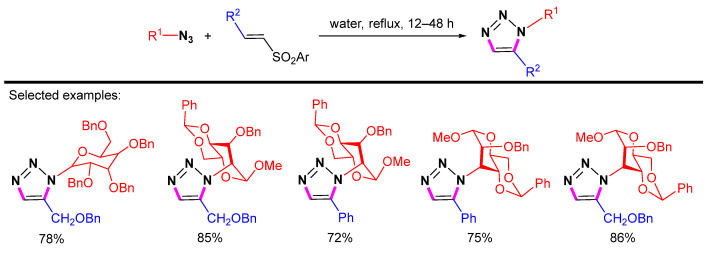
Synthesis of 1,5-disubstituted 1,2,3-triazolylated monosaccharides from azidopyranosides and vinyl sulfones.

Pathak et al. showed that vinyl sulfones reacted with azidopyranosides to produce 1,5-disubstituted 1,2,3-triazoles (Scheme 28) [51]. The reaction was carried out in water at elevated temperatures without any metal catalyst to give 1,5-disubstituted triazolylated monosaccharides in high yields.

**Scheme 29:**
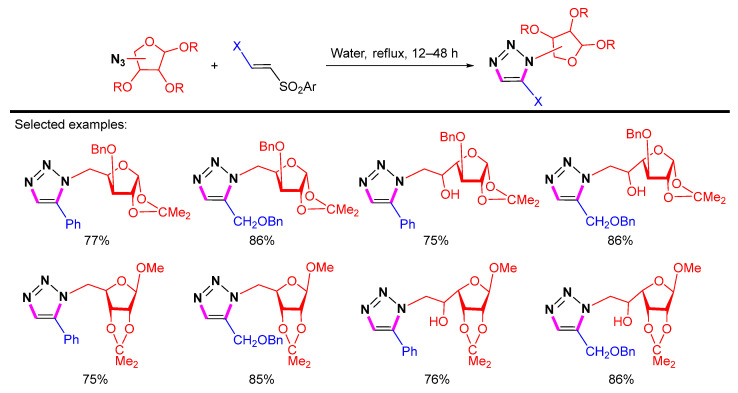
Synthesis of 1,5-disubstituted 1,2,3-triazolylated monofuranosides.

In 2015, they reported that the [3 + 2] cycloaddition reaction of vinyl sulfone derivatives with azides under reflux conditions without metal catalyst could provide 1,5-disubstituted 1,2,3-triazolylated monofuranosides and difuranosides (Scheme 29) [52]. A new possibility for linking furanosides with a stable triazole backbone is offered by the synthesis of these 1,5-disubstituted triazolylated monosaccharides as well as 1,5-disubstituted 1,2,3-triazole-linked disaccharides.

**Scheme 30:**
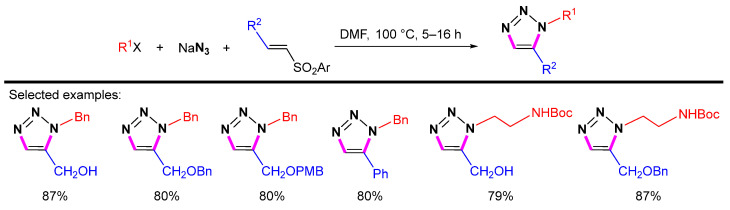
One-pot three-component synthesis of 1,5-disubstituted 1,2,3-triazoles.

Furthermore, Pathak et al. developed the one-pot three-component cycloaddition of vinyl sulfones and sodium azide with various third components, including alkyl bromides, -tosylates, -mesylates or aryl amines, -iodides, to offer a wide variety of 1,5-disubstituted 1,2,3-triazoles (Scheme 30) [53].

#### 4.2.2. Cycloaddition/Elimination of HNO_2_/HOAc Group

**Scheme 31:**
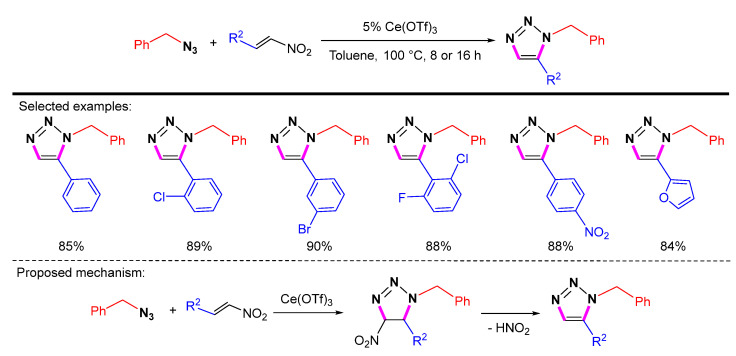
Synthesis of 1,5-disubstituted 1,2,3-triazoles from azides and nitroolefins and proposed mechanism.

In the presence of Ce(OTf)_3_, organic azides undergo [3 + 2] cycloaddition with nitroalkenes. Then the elimination of nitro groups leads to the formation of 1,5-disubstituted 1,2,3-triazoles via aromatization (Scheme 31) [54]. This reaction produces 1,5-disubstituted 1,2,3-triazoles in good to excellent yields with good compatibility for tertiary amines, hydroxyls, and halogens. The advantages of this procedure are the high availability of starting materials, the convenience of the experimental procedure, and the low cost of the catalyst.

**Scheme 32:**
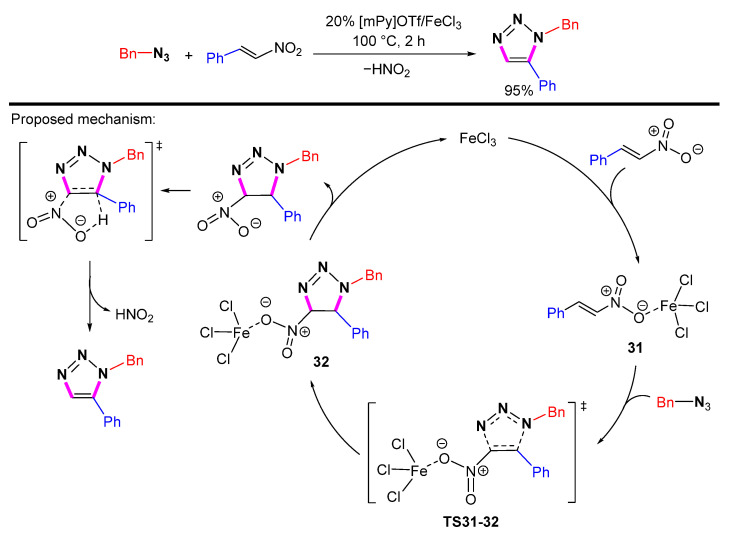
Synthesis of 1,5-disubstituted 1,2,3-triazole by azide-olefin cycloaddition and proposed mechanism.

In 2018, Maiuolo et al. reported the preparation of 1,5-disubstituted 1,2,3-triazole derivatives via FeCl_3_-mediated azide-olefin cycloaddition in ionic liquids (Scheme 32) [55]. DFT calculations indicate that the first step of the reaction is the coordination of FeCl_3_ with the nitroolefin compound to form an activated intermediate **31**. Intermediate **31** reacts with the azide to produce the triazoline intermediate via the transition state **TS31-32**. The last step eliminates HNO_2_ to give 1,5-disubstituted 1,2,3-triazoles.

**Scheme 33:**
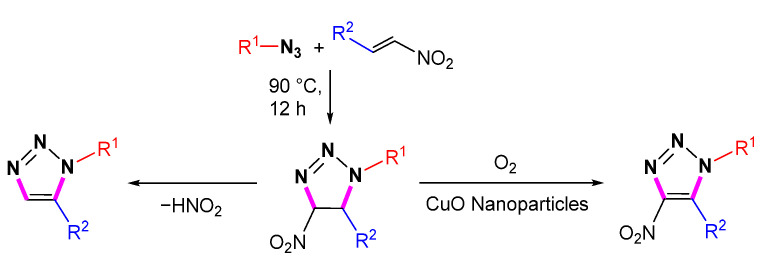
The eliminative azide-olefin cycloaddition and oxidative azide-olefin cycloaddition of azides and nitro alkenes.

Elangovan et al. described the synthesis of 1,2,3-triazoles through [3 + 2] cycloaddition under solvent-free and catalyst-free conditions (Scheme 33) [56]. The triazolines formed from the cycloaddition of azides and olefins are unstable. The aromatized 1,2,3-triazoles are obtained by the elimination of HNO_2_ in the absence of a catalyst. The aromatic stability of the product and the good leaving ability of the NO_2_ group are the main driving forces.

**Scheme 34:**
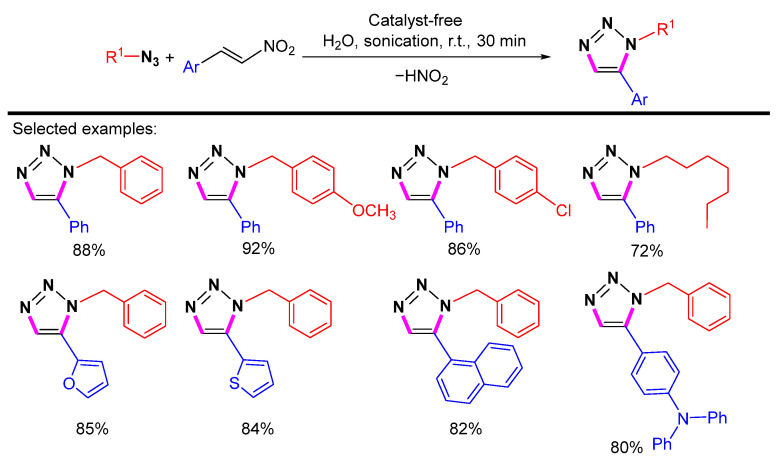
Synthesis of 1,5-disubstituted 1,2,3-triazole from azides and nitro alkenes.

In 2021, Karthikeyan et al. reported a simple and efficient catalyst-free cycloaddition for the preparation of 1,5-disubstituted 1,2,3-triazoles from azides and nitro alkenes in an aqueous base (Scheme 34) [57]. This cycloaddition proceeds under ultrasound irradiation with the scope of broad substrates, simple work-up, and high regioselectivity.

**Scheme 35:**
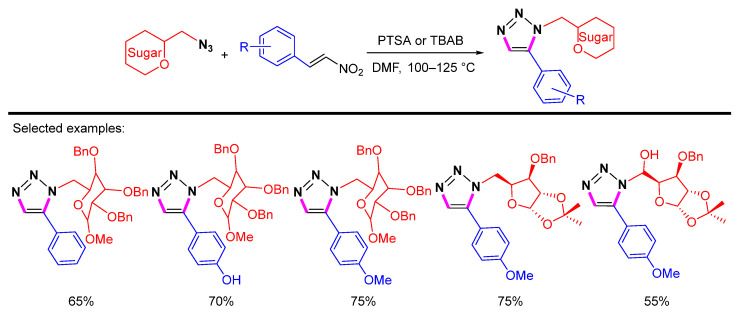
Synthesis of 1,5-disubstituted triazolyl glycoconjugates from glycosyl azides with nitro alkenes.

The generation of 1,5-substituted 1,2,3-triazoles through eliminating NO_2_ groups is an important method for the synthesis of sugar scaffolds. In 2016, Tiwari et al. presented a cycloaddition for the synthesis of 1,5-disubstituted triazolyl glycoconjugates from different glycosyl azides with nitro-olefins with phase transfer catalysts, e.g., *p*-toluenesulfonic acid (PTSA), tetrabutylammonium bromide (TBAB) (Scheme 35) [58].

**Scheme 36:**
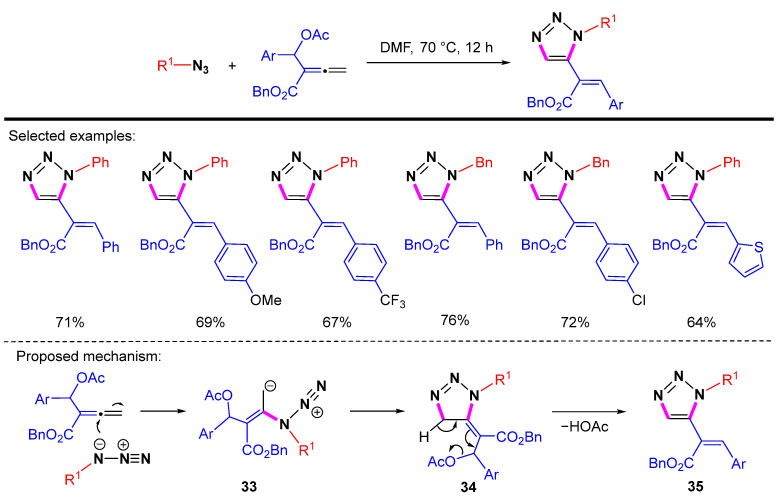
[3 + 2]-Cycloaddition of β-acetoxy allenoates with azides and proposed mechanism.

In 2021, Swamy et al. reported the cycloaddition/cyclization reactions for the synthesis of 1,5-disubstituted 1,2,3-triazoles under metal-free conditions using β-acetoxy allenoates [59]. A plausible pathway for the formation of 1,2,3-triazoles is shown in Scheme 36. The allyl/alkyl nitrogen atom first attacks the β-position of the allenoates to give the intermediate **33**. Next, **33** undergoes an intramolecular addition to give the cycloaddition adduct **34**. Finally, acetic acid is eliminated from intermediate **34** to form the final product **35**.

#### 4.2.3. Cycloaddition/Elimination of Protection Group

**Scheme 37:**
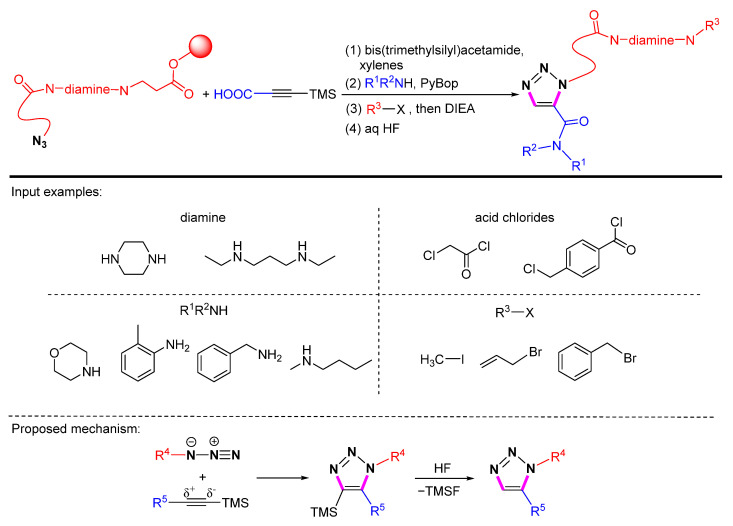
Solid-phase 1,5-selective cycloaddition of azides immobilized on REM resin with 1-trimethylsilylacetylene.

Generally, 1,5-selective cycloaddition has limited use in solid-phase synthesis and drug discovery for the synthesis of 1,2,3-triazoles. In 2004, Hlasta et al. found that 1-trimethylsilylacetylene can react with azides immobilized on REM resin in a [3+2] cycloaddition to yield the corresponding 1,4,5-substituted 1,2,3-triazoles [60]. The TMS group can be removed by contacting with 10 equiv of HF (50% aq) in THF for 4 h at room temperature to produce the 1,5-substituted 1,2,3-triazoles (Scheme 37). A small library (2 × 2 × 4 × 3) of 1,5-substituted 1,2,3-triazoles with an average purified yield of 68% was established through the cycloaddition of azides on REM resin with acetylene.

**Scheme 38:**
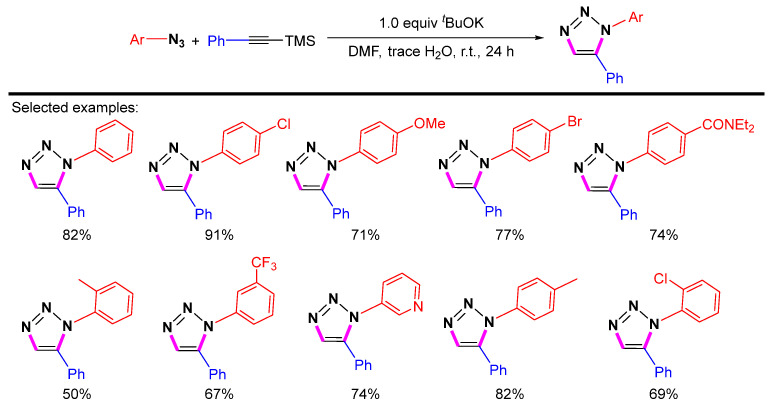
Synthesis of 1,5-disubstituted 1,2,3-triazoles from azides and TMS-alkynes.

In 2012, Lin et al. studied the synthesis of 1,5-disubstituted 1,2,3-triazoles through direct desilylation of TMS-alkynes (Scheme 38) [61]. The use of *^t^*BuOK as a desilylating reagent results in the regioselective formation of 1,5-disubstituted 1,2,3-triazoles. When a trace of water is added, this cycloaddition has good yields at room temperature. In this process, desilylation and subsequent cycloaddition are necessary.

**Scheme 39:**
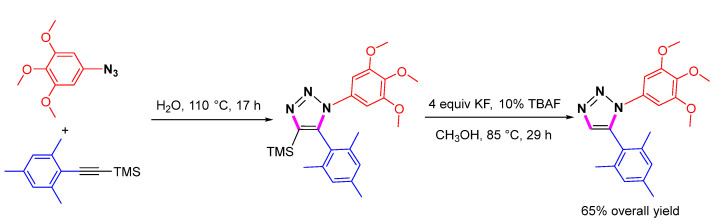
Synthesis of 1,5-disubstituted 1,2,3-triazoles by one pot cycloaddition/desilylation.

Another synthetic route to 1,5-disubstituted 1,2,3-triazoles with high efficiency and regioselectivity was developed using a thermal dipolar cycloaddition reaction between trimethylsilylacetylenes and azides (Scheme 39) [62]. The TMS-modified 1,2,3-triazoles can be desilylated using potassium fluoride and catalytic amounts of tetrabutylammonium fluoride (TBAF) in a methanol solution.

**Scheme 40:**
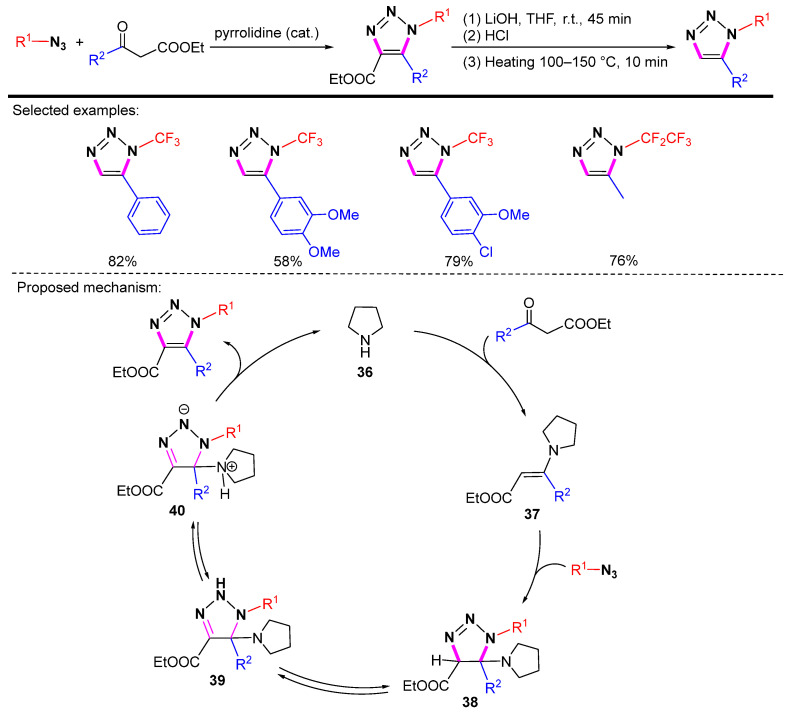
Synthesis of 1,5-disubstituted 1,2,3-triazoles via hydrolysis and decarboxylation and proposed mechanism.

Highly functionalized *N*-perfluoroalkyl-1,2,3-triazoles were efficiently synthesized from azidoperfluoroalkanes [63]. Enamine **37** generated in situ could easily participate in the azidecarbonyl [3 + 2] cycloaddition reaction, providing a simple method for the synthesis of triazole frameworks with good to excellent yields. The basic hydrolysis and decarboxylation of the ethoxycarbonyl-substituted 1,2,3-triazoles can form 1,5-disubstituted 1,2,3-triazoles in high yields (Scheme 40). The proposed mechanism can be summarized as follows. Azide and enamine **37** undergo cycloaddition to form triazoline intermediate **38**. After the 1,3-hydrogen shift, **38** rearranges to give **40**. One molecule of amine is then eliminated to complete the catalytic cycle.

### 4.3. Formal 1,5-Selective Click Reaction of Azide with Alkyne via Wittig Reaction

**Scheme 41:**
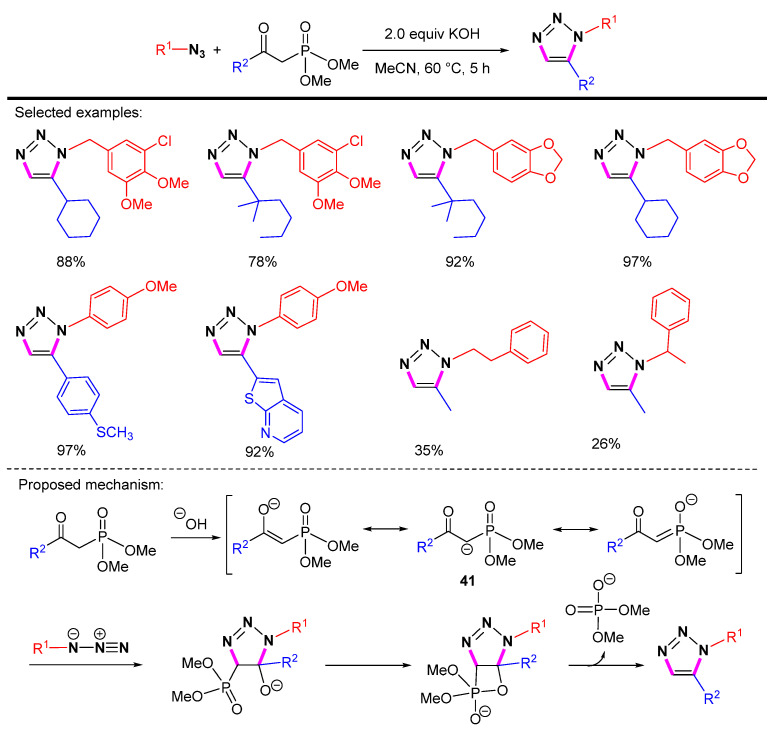
Azide-enolate [3 + 2] cycloaddition and proposed mechanism.

In 2016, Carlos et al. developed a simple and efficient method for the 1,5-selective cycloaddition of azides with β-ketophosphonates (Scheme 41) [64]. The scope and diversity of this protocol include the effective synthesis of 1-alkyl-substituted, 1-aryl-substituted, 5-alkyl-substituted, and 5-aryl-substituted 1,2,3-triazoles. The phosphoryl-stabilized carbanion **41** is able to couple with the azide in a highly regioselective manner to form the corresponding oxaphosphetane. Washing with water makes it easy to separate the desired products as well as a free phosphate by-product.

**Scheme 42:**
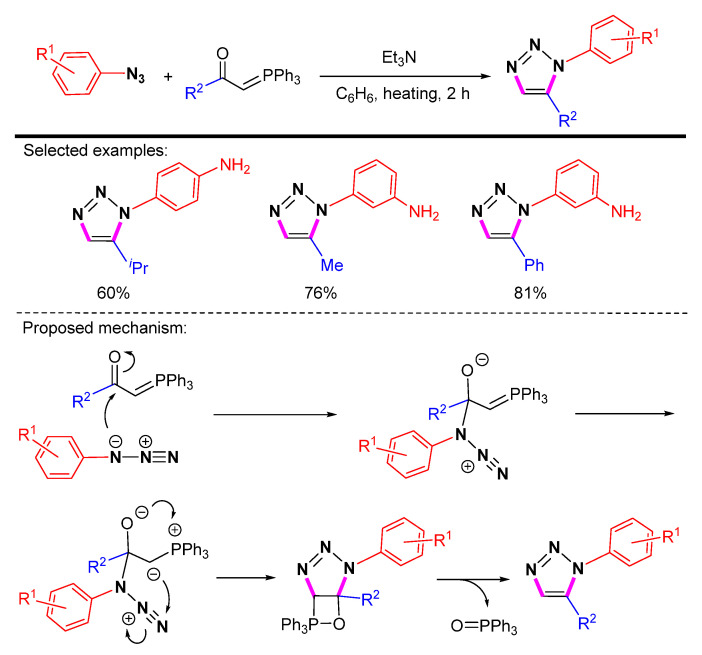
Synthesis of 1,5-disubstituted 1,2,3-triazoles by eliminating phosphorus-containing compounds and proposed mechanism.

Obushak et al. found that the reaction of aryl azides with phosphorus ketoylides was a convenient method for the synthesis of 1,5-disubstituted 1,2,3-triazoles (Scheme 42) [65]. The 1,5-disubstituted 1,2,3-triazoles could be obtained by eliminating phosphorus-containing compounds in near quantitative yields.

### 4.4. Other Formal 1,5-Selective Click Reaction of Azide with Alkyne

**Scheme 43:**
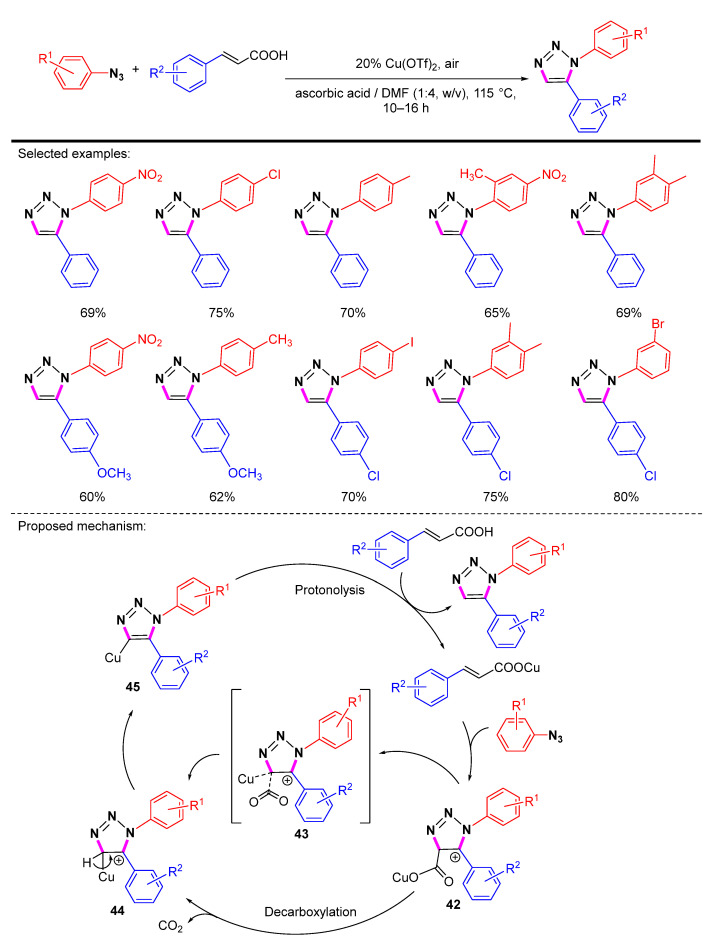
Synthesis of 1,5-disubstituted 1,2,3-triazoles via Cu-catalyzed decarboxylation.

Kumar et al. presented the first evidence that Cu(II) can catalyze the synthesis of 1,5-disubstituted 1,2,3-triazoles via the coupling of benzoyl derivatives and substituted styryl carboxylic acids (Scheme 43) [66]. The first step of the reaction is the regioselective cyclization of azide with cinnamic acid to form the cationic intermediate **42**. Intermediate **42** undergoes a decarboxylation reaction to produce copper triazolide **44**, which subsequently loses a proton to give a copper complex of 1,4,5-trisubstituted 1,2,3-triazoles. In acidic media, the copper complex **45** readily undergoes proton decomposition to produce 1,5-disubstituted 1,2,3-triazoles and the Cu(II) species to complete the catalytic cycle. They believed that the Cu(II) species could be regenerated from the Cu(I) species with oxygen under acidic conditions. However, in the proposed mechanism, the origin of Cu(I) at the very beginning and how air was involved in the catalytic cycle as an oxidant is not mentioned and is still unclear.

**Scheme 44:**
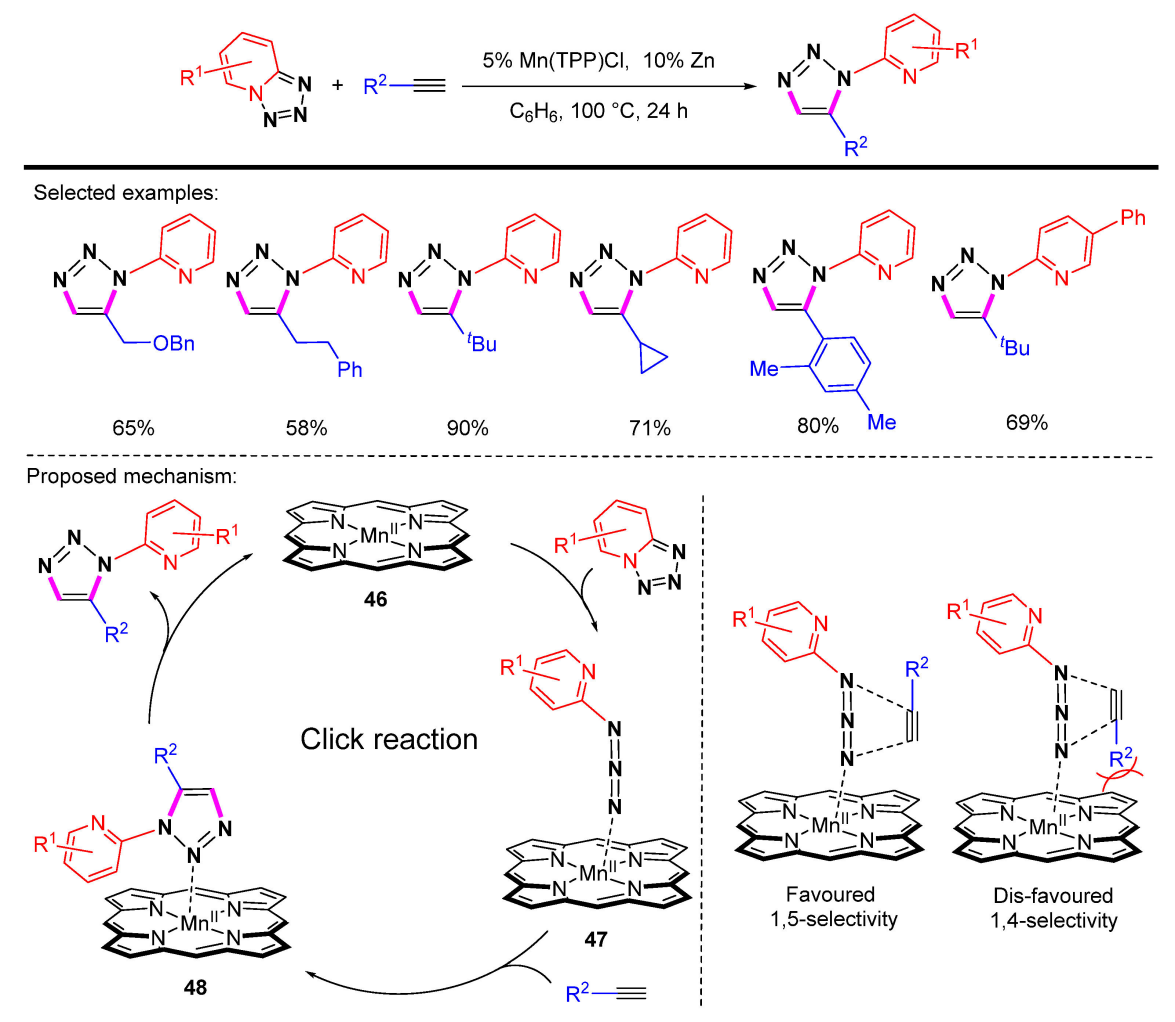
Synthesis of pyridyl-substituted 1,5-disubstituted 1,2,3-triazoles by Mn-porphyrin catalyzed cycloaddition of tetrazoles with terminal alkynes and proposed mechanism.

Chattopadhyay et al. reported the synthesis of pyridyl-substituted 1,5-disubstituted 1,2,3-triazoles by Mn-porphyrin catalyzed cycloaddition of tetrazole with terminal alkynes (Scheme 44) [67]. The method is compatible with a wide range of substrates for the reactions. A possible mechanism was proposed. Mn-bound complex **46** coordinates with azide to form intermediate **47**. Intermediate **47** readily undergoes a cycloaddition reaction in the presence of terminal alkyne to produce intermediate **48**, which then releases the click product and regenerates the active species **46**. The source of the 1,5-selectivity generated in this reaction is the effect of steric hindrance.

## 5. Conclusions

In this context, we briefly described the discovery and overviews of 1,5-selective click chemistry for the synthesis of 1,5-disubstituted 1,2,3-triazoles via metallacyclic intermediate, acetylide intermediate, or the elimination of substituents. There has been growing interest in 1,5-disubstituted 1,2,3-triazoles, and chemists have attempted and developed a number of processes that can effectively control the regioselectivity of the cycloaddition reaction by utilizing metal catalysts or controlling the substituent steric hindrance and electronic effect.

The 1,5-selective [3 + 2] cycloaddition of azides with alkynes is still an area that has not yet been fully developed. Many interesting problems are still waiting to be solved. The involvement of organometallic reagents can, of course, be effective in the rapid generation of anions, but there are problems, such as insufficient compatibility of functional groups or harsh reaction conditions unsuitable for chemical biology studies as well as metal residues. With the development of DFT theoretical calculations, more and more scientists are focusing on understanding reaction mechanisms and sources of regioselectivity, but the mechanisms are still unclear for most currently reported reactions.

The impact of 1,5-selective click chemistry is increasing tremendously day by day, not only in the field of organic synthesis but also in drug discovery efforts, polymer chemistry, and in different disciplines of material science. The cycloaddition reactions forming 1,5-disubstituted 1,2,3-triazoles have found extremely successful applications in the synthesis of nanostructures, protein conjugates, and polymeric materials due to their regiospecificity and unique chemoselectivity. This provides more possibilities for building functionalized and well-defined macromolecules and nanostructures that will be used in more areas.

## Data Availability

Not applicable.
